# miR-27b regulates myogenic proliferation and differentiation by targeting *Pax3* in goat

**DOI:** 10.1038/s41598-018-22262-4

**Published:** 2018-03-02

**Authors:** Ying-Hui Ling, Meng-Hua Sui, Qi Zheng, Kang-Yan Wang, Hao Wu, Wen-Yong Li, Yong Liu, Ming-Xing Chu, Fu-Gui Fang, Li-Na Xu

**Affiliations:** 1 College of Animal Science and Technology, Anhui Agricultural University, Anhui Hefei, China; 2 Local animal genetic resources conservation and biobreeding laboratory of Anhui province, Anhui Hefei, China; 3 Institute of Plant Protection and Agro-Products Safety, Anhui Academy of Agricultural Sciences, Hefei, Anhui 230031 China; 4 Key Laboratory of Embryo Development and Reproductive Regulation of Anhui Province, Fuyang Normal University, Fuyang, Anhui 236037 China; 5 Key Laboratory of Farm Animal Genetic Resources and Germplasm Innovation of Ministry of Agriculture, CAAS, Beijing, 100193 China

## Abstract

This study found that miR-27 is expressed in muscle and regulates muscle proliferation and differentiation. We explored the function and regulatory mechanism of miR-27b in goat muscle proliferation and differentiation. Compared with the Boer goat, higher expression of miR-27b was observed in all of the collected muscle tissues of Anhuai goat, excluding the kidney, whereas the opposite expression pattern was observed for *Pax3*, which showed lower expression in Anhuai goat. Expression of miR-27b decreased gradually during the proliferation of skeletal muscle satellite cells in Anhuai goat and increased during differentiation; however, the expression pattern of *Pax3* was opposite. The regulatory activity of miR-27b demonstrated that miR-27b inhibited the proliferation of skeletal muscle satellite cells, but promoted their differentiation. Moreover, function research demonstrated that *Pax3* negatively regulated myogenic differentiation of goat skeletal muscle satellite cells, but accelerated their proliferation. The results of a dual-luciferase reporter analysis showed that miR-27b directly targeted the 3’-untranslated regions of *Pax3* mRNA, and western blot and immunofluorescence staining analyses showed that miR-27b inhibited expression of the Pax3 protein. In goats, miR-27b can regulate myogenic proliferation and differentiation by targeting *Pax3*.

## Introduction

The goat is an important economic animal in China, and the consumption of mutton is increasing each year. Therefore, the breeding of high quality meat goat is an urgent requirement for production. Myogenesis is an important process of tissue formation, which affects meat quality and yield, and myogenesis is regulated by the sequential expression of myogenic regulatory factors (MRFs), including myogenic determination factor *MyoD*, *Myf5* and *MyoG*^1,2^. More recently, it has been shown that *Pax3* and its close homolog, *Pax7*, are expressed in a population of myogenic precursor cells that give rise to a population of adult muscle stem cells^3,4^. Moreover, cells that fail to express *Pax3* or *Pax7* die or switch to another cell fate^4^. *Pax3* is a member of the Paired Box transcription factor family^5–7^ and has essential functions in myogenesis, as revealed by the discovery that compound *Pax3* and *Myf5* mutant embryos completely lack trunk muscles and do not express *MyoD*^7^. The myogenic determination genes, *Mrf5* and *MyoD*, are direct targets of *Pax3*^8,9^, which direct progenitors into the myogenic programme. During postnatal myogenesis, *Pax3* is transiently expressed during muscle stem cell (“satellite cell”) activation in a highly proliferative intermediate progenitor cell population^10^.

Myogenesis is also regulated by a number of transcription factors and several noncoding RNAs, which act at specific steps, including the commitment of progenitor (satellite) cells to myogenic lineage and myoblast proliferation, differentiation, and fusion^11,12^. miRNAs comprise a class of non-coding RNAs that suppress mRNA translation or induce degradation, and it plays a critical role in many processes, such as cell proliferation and differentiation, among others. To date, many muscle-specific miRNAs, miR-1, miR-133, miR-206, miR-27, were found, which have been shown to play critical roles in skeletal muscle development. miR-1 and miR-206 promote the differentiation of myoblasts, and miR-133 promotes satellite cell proliferation^13–15^. As a member of the miR-27 family, miR-27b plays an important role in muscle development. miR-27b can inhibit skeletal muscle satellite cell proliferation but promote its differentiation^16^. In addition, miR-27b can regulate Pax3 protein levels, and this downregulation ensures rapid and robust entry into the myogenic differentiation programme in mice^16^. However, these studies were all discovered in mouse, human or bovine, and few experiments describe the potential role of miRNAs in goat skeletal muscle satellite cells.

Anhuai goat is a native breed to China that is well known for its high prolificacy and good skin quality, however, it grows slowly and has a small size. Therefore, improving the performance of meat production is a primary objective of the improved breeding of Anhuai goat. miR-27b has been found to be expressed in a tissue-specific manner in the muscle of Anhuai goat^17^, however, its functions and mechanisms remain unclear. In this article, we analysed the expression patterns of miR-27b and *Pax3* in different muscle tissues of goat and studied the potential of miR-27b as a regulator of *Pax3* in Anhuai goat. miR-27b targets the 3′-UTR of *Pax3* and affects the post-transcriptional expression of *Pax3*, with consequences for the onset of skeletal muscle differentiation.

## Results

### Isolation, culture and myogenic differentiation of goat skeletal muscle satellite cells

**Figure 1:**
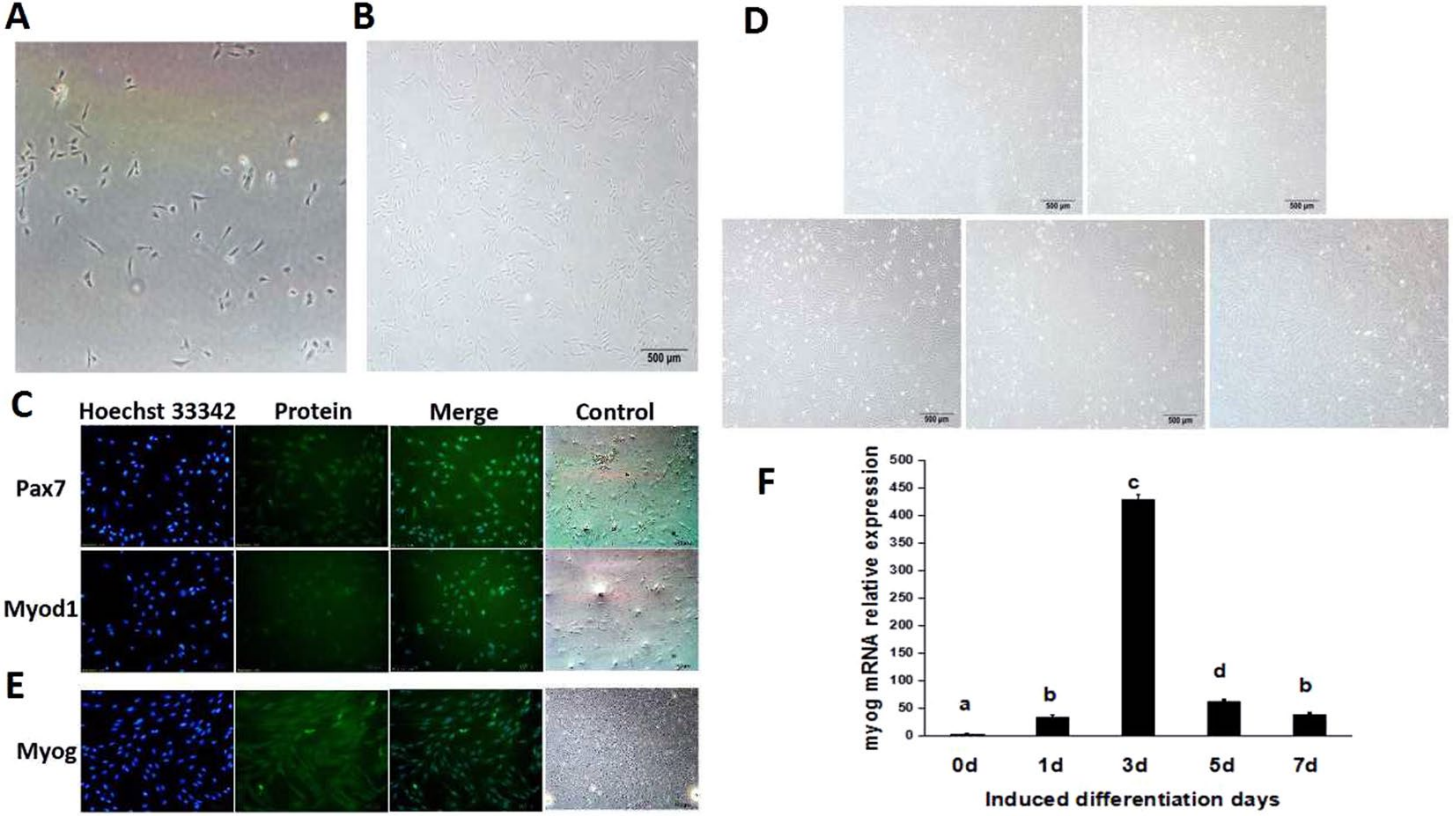
Isolation, identification and myogenic differentiation of goat skeletal muscle satellite cells. (**A**) Four-day cultures of isolated goat satellite cells (40x). (**B**) Sixth generation of goat satellite cells (40x). (**C**) Immunofluorescence detection of Pax7 and MyoD1 in goat satellite cells. (**D**) Morphological observation of induced different numbers of days of goat satellite cells (40x). (**E**) Immunofluorescence detection of Myog in goat satellite cells. (**F**) Relative *Myog* mRNA expression. The fold changes were analysed using the 2^−△△Ct^ method. The data are presented as the mean ± standard error (n = 3). Values with different letters were significantly different (P < 0.05).

The original isolated cells were small and adherent (Fig. 1). As the cells were subcultured and purified, the cell volume became larger and the morphology tended to be stable, with a predominantly fused shape (Fig. 1). The 6th generation cells were selected for identification. The results of immunofluorescence staining showed Pax7 and Myod1 positivity in the isolated cells (Fig. 1), which proved that the satellite cells were successfully isolated and had a high purity. Cell myogenic differentiation was induced at a cell density of 80~90%. The morphological changes of cells induced on days 0, 1, 3, 5 and 7 were observed using an inverted microscope. The results showed that as the days of induction increased, the cells gradually began to differentiate and fuse with each other into myotubes and to exhibit a certain direction (Fig. 1). Immunofluorescence staining and qPCR detection of the expression of the satellite cell differentiation marker gene *Myog* were positive (Fig. 1). The above results showed that the satellite cells obtained in this study had superior myogenic differentiation ability.

### Expression of *Pax3* is negatively related to miR-27b during goat muscle development

**Figure 2:**
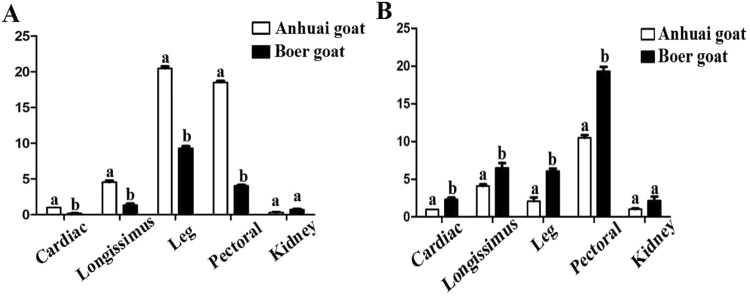
Expression of miR-27b and *Pax3* in the different muscle tissues of Boer goats and Anhuai goats. (**A**) The expression of miR-27b in the two goat breeds. (**B**) The expression of *Pax3* in the two goat breeds. The fold changes were analysed using the 2^−△△Ct^ method, with corrections for PCR efficiency. The bars indicate the standard error of values from three separate experiments. The data are presented as the mean ± standard error (n = 3). Values with different letters were significant difference (P < 0.05).

First, the total RNA was successfully extracted from all the collected tissues and cells, and then the expression pattern of *Pax3* and miR-27b in the different goat breeds, muscle tissues, and cells were detected using qPCR. Compared with the Boer goat, the expression of miR-27b was higher in all the collected muscle tissues of Anhuai goat, excluding kidney, whereas the expression of *Pax3* was opposite, demonstrating lower values in all tissues in Anhuai goat (Fig. 2). In addition, the expression of miR-27b was highest in the leg muscle of the two goat breeds, and the expression of *Pax3* was highest in breast muscle (Fig. 2).

**Figure 3:**
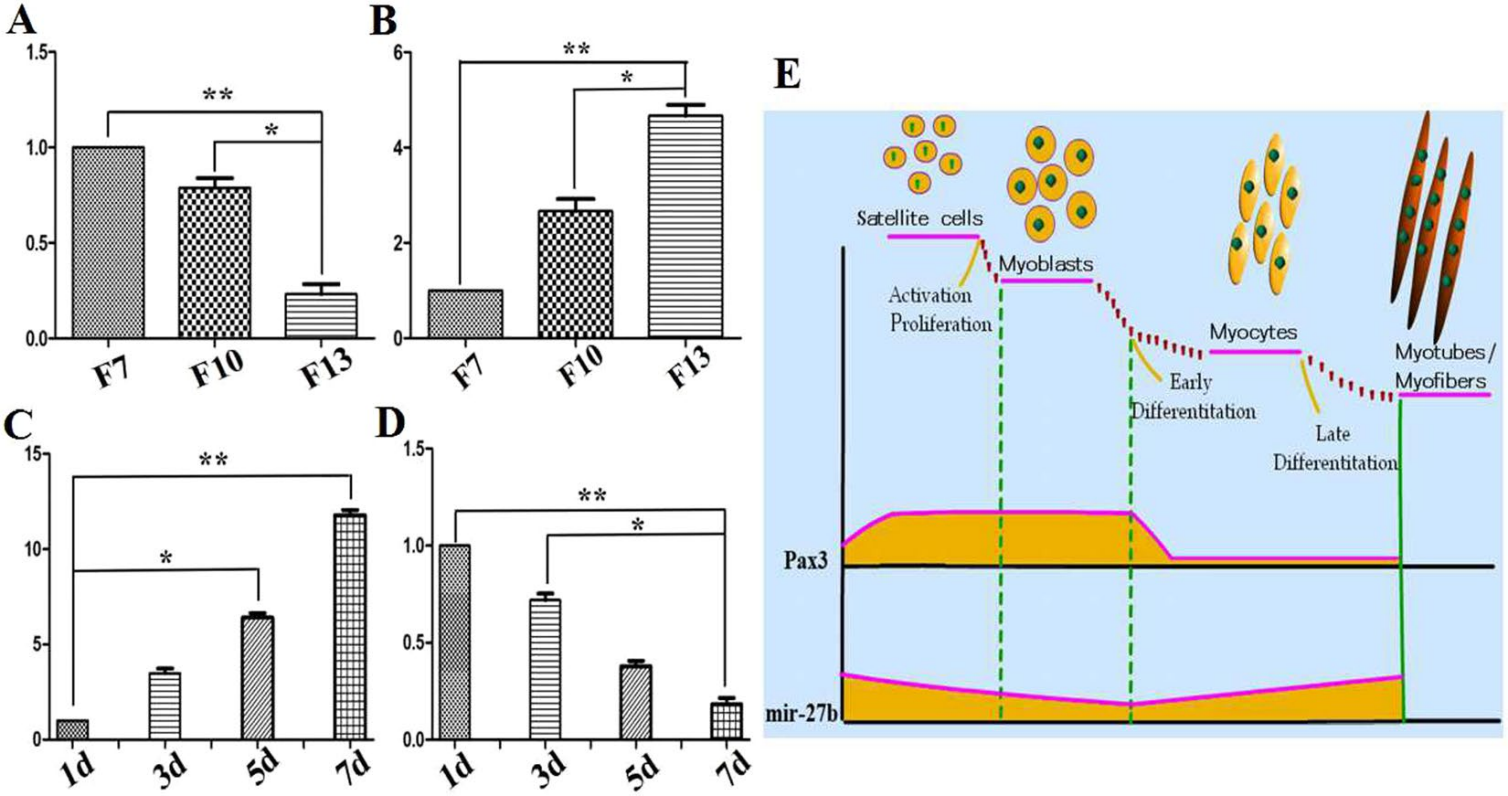
Expression of miR-27b and *Pax3* during the differentiation and proliferation of satellite cells. (**A**) The expression of miR-27b during proliferation. (**B**) The expression of *Pax3* during proliferation. (**C**) The expression of miR-27b during differentiation. (**D**) The expression of *Pax3* during differentiation. (**E**) Schematic representation of the expression pattern of miR-27b and *pax3* during muscle differentiation. The fold changes in expression were analysed using the 2^−△△Ct^ method with corrections for PCR efficiency. The bars indicate the standard error of values from three separate experiments. The data are presented as the mean ± standard error (n = 3). Values with *were significantly different (*means P < 0.05; **means P < 0.01). F7, F10 and F13 in the figure represent the 7th, 10th and thirteenth generations, while 1 d, 3 d, 5 d and 7 d represent 1 day, 3 days, 5 days and 7 days, respectively.

To further elucidate the relationship of miR-27b and *Pax3* during the development of muscle satellite cells, the expression of the two transcripts in the cells, which are in different stages of proliferation and on different days of differentiation, were analyzed. The results showed that the endogenous expression of miR-27b decreased during the proliferation of satellite cells; however, the expression of *Pax3* gradually increased. Moreover, the expression of miR-27b increased, and the *Pax3* decreased during the differentiation of goat skeletal muscle satellite cells (Fig. 3). The results indicated that the expression of *Pax3* was negatively related to miR-27b during goat muscle development, and miR-27b could inhibit proliferation and promote the differentiation of the satellite cells in goat by regulating the expression of *Pax3*.

### The function of miR-27b is to inhibit proliferation and promote goat skeletal muscle satellite cell differentiation

**Figure 4:**
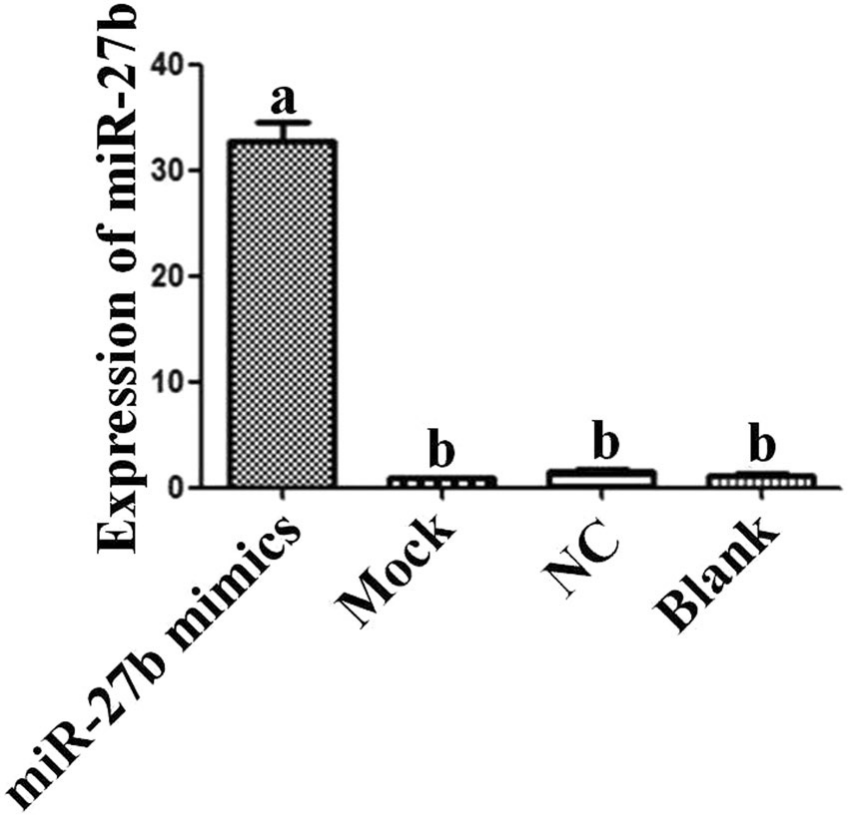
Expression of miR-27b in cells after transfection.

**Figure 5:**
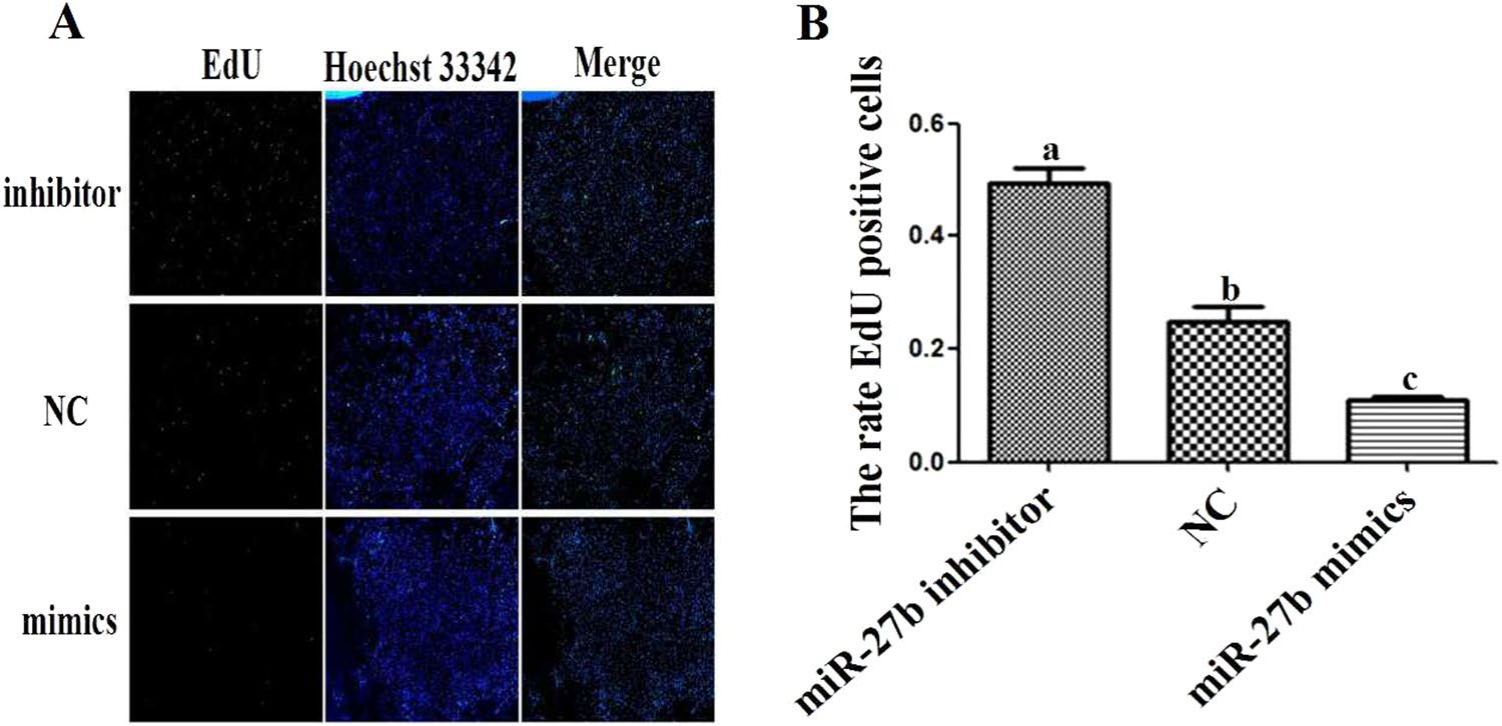
Effect of miR-27b on the proliferation of satellite cells. (**A**) Proliferation SSC labelled with EdU, where green shows EdU-positive cells and blue shows cell nuclei. (**B**) The percentage of EdU-positive cells (bars with different letters are significantly different, *p* < 0.05).

Transfect the miR-27b mimics, miR-27b inhibitor and miR-27b Negative Control (NC) into skeletal muscle satellite cells and qPCR was used to detect the expression of miR-27b in the cells after transfection to validate the transfection efficiency. Compared with NC, the expression of miR-27b was successfully influenced by transfection (Fig. 4). Furthermore, the EdU incorporation assay was performed to detect the influence of miR-27b on the proliferation of goat skeletal muscle satellite cells. Compared with the NC, the percentage of EdU-positive cells transfected with miR-27b mimics was significantly decreased, and the proliferation of the cells was decreased by 43%. However, the percentage of EdU-positive in the cells transfected with miR-27b inhibitor was significantly increased, and the proliferation of the cells was increased by 35% (Fig. 5). The results illustrated that miR-27b can inhibit goat skeletal muscle satellite cell proliferation.

**Figure 6:**
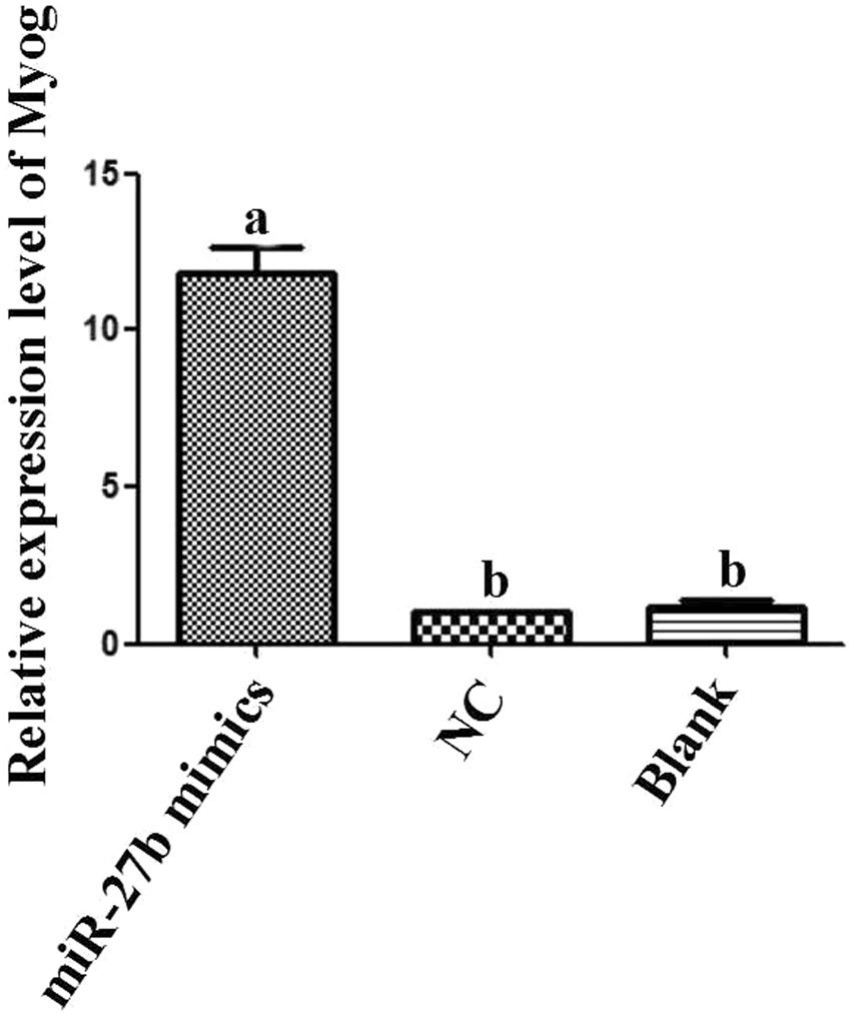
Expression of *MyoG* mRNA in satellite cells. Bars with different letters are significantly different, *p* < 0.05.

The *MyoG* gene is a marker gene for differentiation initiation of skeletal muscle satellite cells. We detected the expression pattern of *MyoG* in satellite cells transfected with miR-27b mimics by qPCR. Compared with the NC or blank, the expression of *MyoG* was significantly increased in response to miR-27b mimic treatment, which indicated that miR-27b could promote the differentiation of satellite cells (Fig. 6).

### *Pax3* inhibits the differentiation, but accelerates the proliferation, of goat skeletal muscle satellite cells

**Figure 7:**
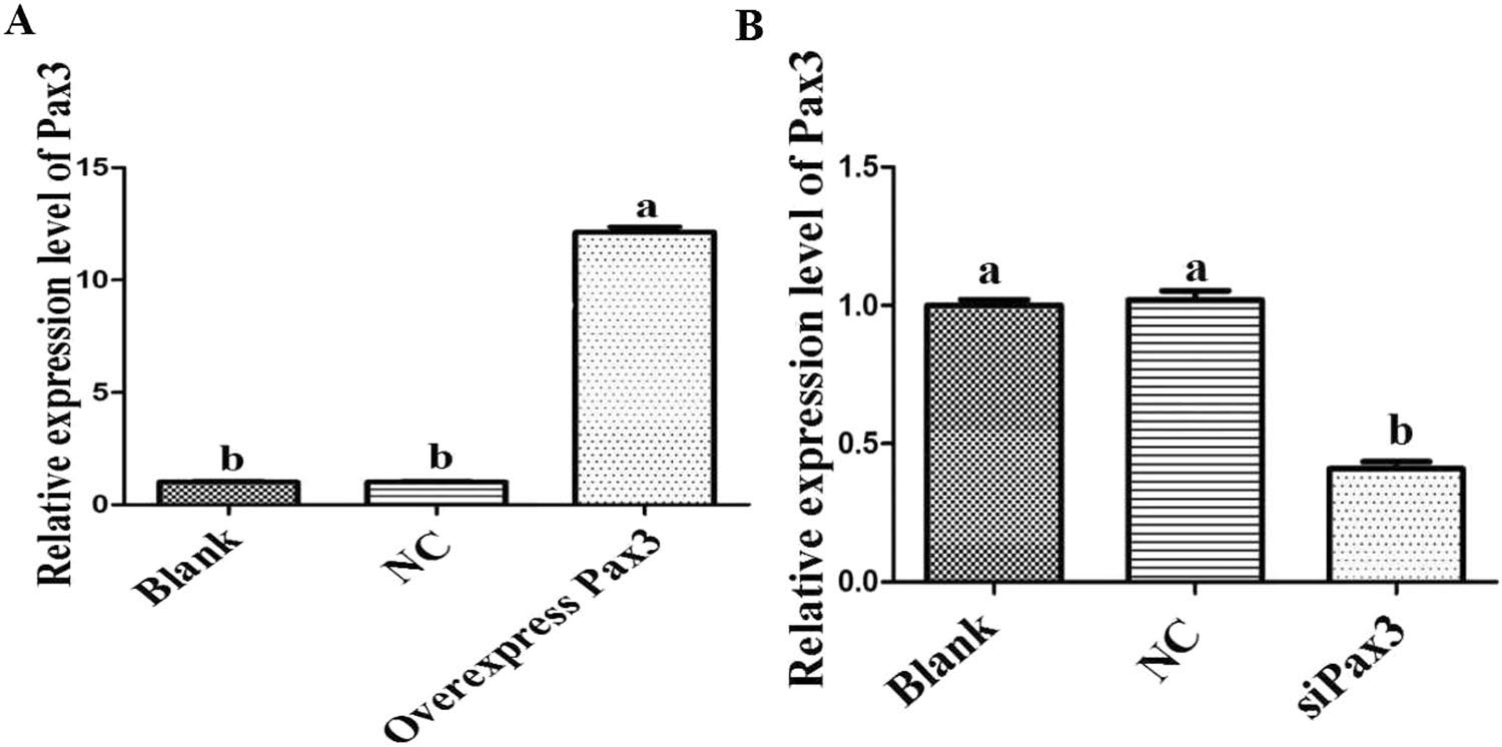
Expression of *Pax3* in cells transfected with siRNA or over-expression vector. (**A**) The expression of *Pax3* in the cells transfected with over-expression vector. (**B**) The expression of *Pax3* in cells transfected with siRNA. Bars with different letters are significantly different, *p* < 0.05.

**Figure 8:**
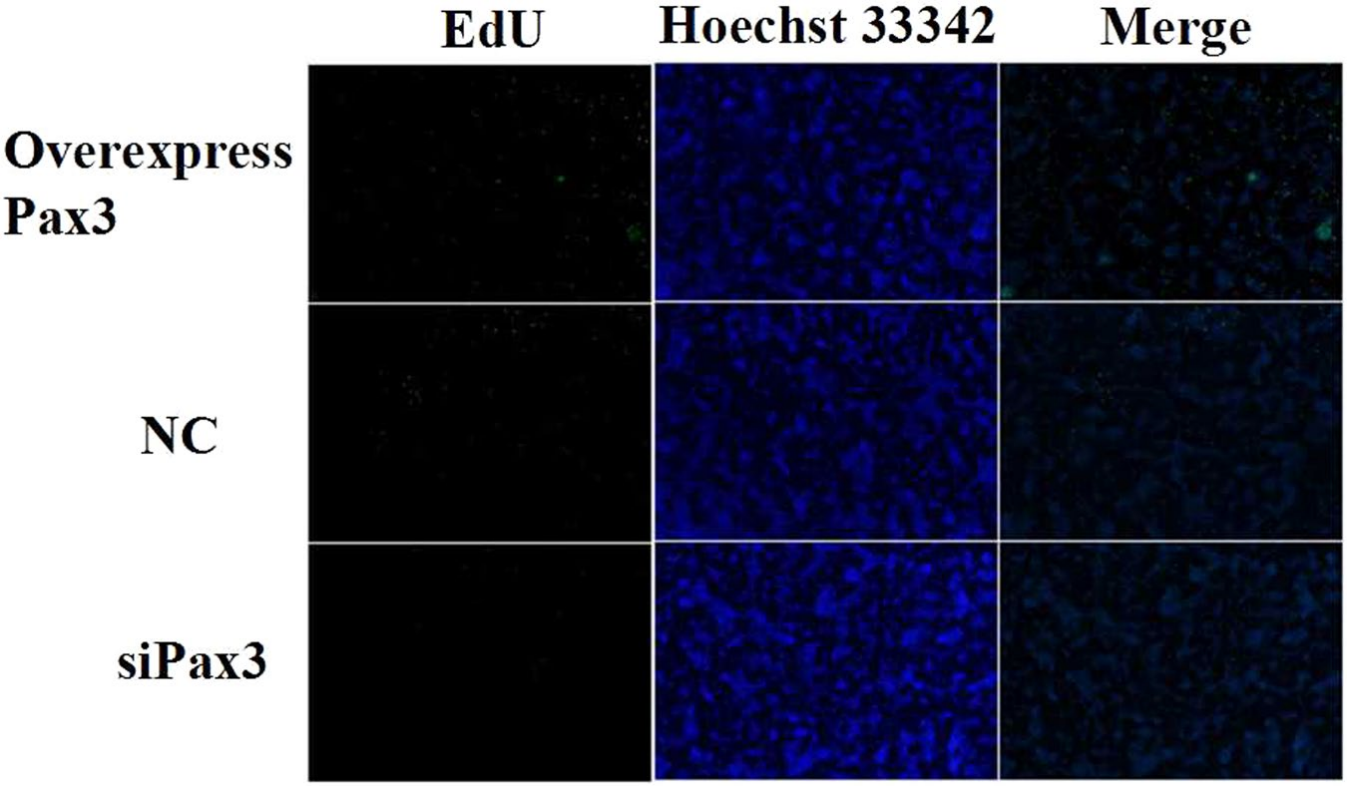
Effect of *Pax3* on the proliferation of satellite cells.

**Figure 9:**
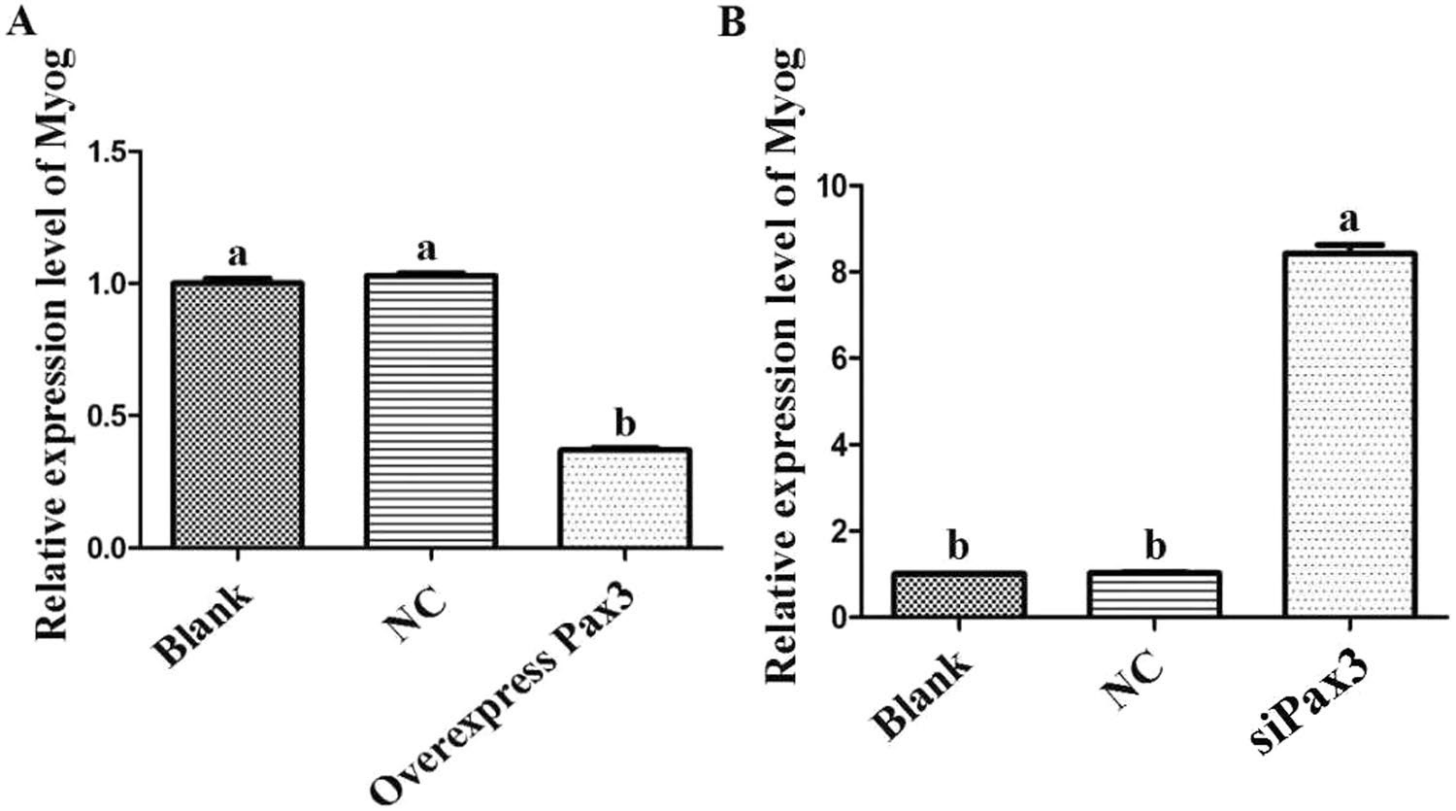
Expression of *Myog* in cells transfected with siRNA or *Pax*3 over-expression vector. (**A**) Expression of *Myog* in cells transfected with over-expression vector. (**B**) Expression of *Myog* in cells transfected with siRNA. Bars with different letters are significantly different, *p* < 0.05.

To examine the role of *Pax3* in the development of goat muscle, we successfully silenced or enhanced the expression of *Pax3* in goat skeletal muscle satellite cells through siRNA or constructed over-expression vector application (Fig. 7), and we detected a variety of differentiation and proliferation effects on the cells. Compared with the NC, there were significantly more EdU-positive cells in the *Pax3* up-regulated group and significantly fewer in the *Pax3* down-regulated group (Fig. 8). The expression levels of *MyoG*, which is a marker gene for cell differentiation, were detected to analyse the influence of *Pax3* on skeletal muscle satellite cells. The results showed that over-expression of *Pax3* resulted in the down-regulation of the *MyoG* gene, while knockdown of *Pax3* increased the expression of *MyoG* (Fig. 9). Thus, we inferred that *Pax3* could inhibit the differentiation but accelerate the proliferation of muscle satellite cells in goat.

### miR-27b inhibits the post-transcriptional expression of *Pax3* in goat skeletal muscle satellite cells

**Figure 10:**
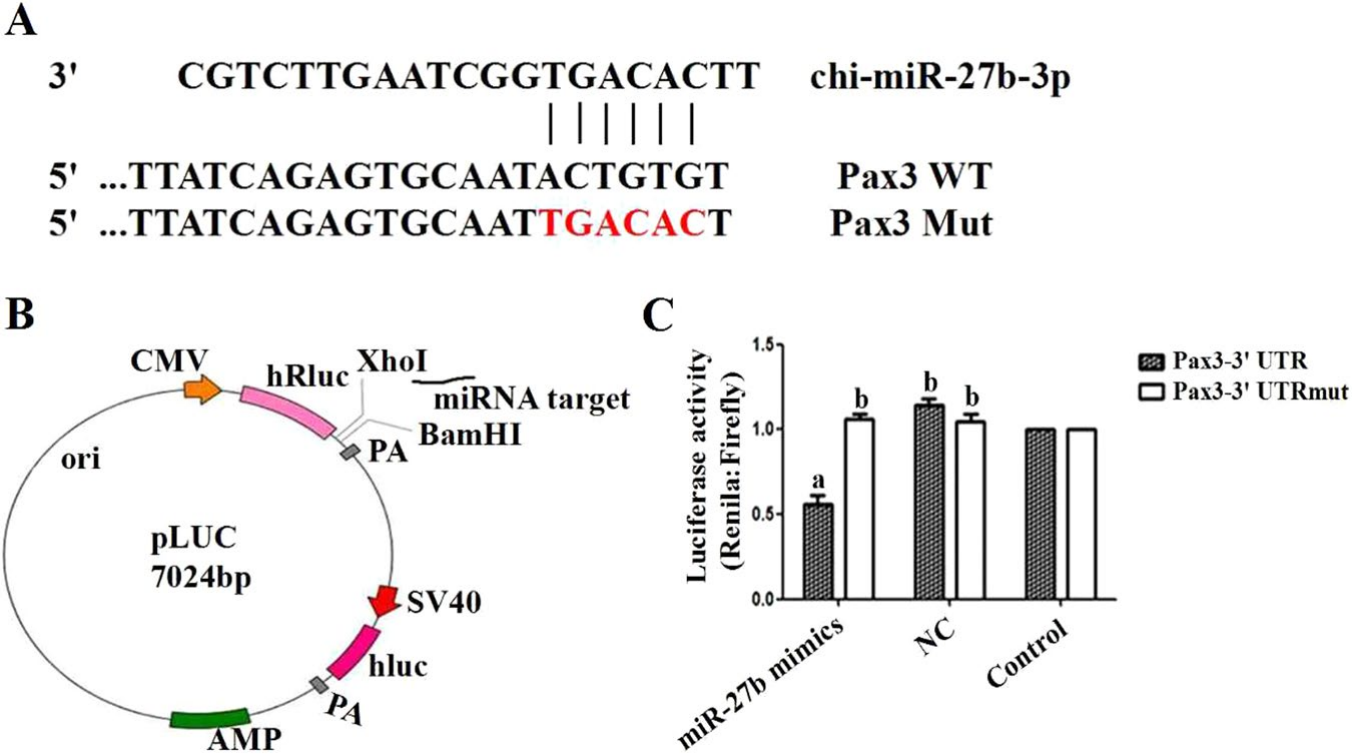
*Pax3* is a target gene of miR-27b. (**A**) Predicted miR-27b target site in the 3′UTR of goat *Pax3*. (**B**) Information for the dual-luciferase vector. (**C**) Pooled miR-27b mimics and miR-27b NC were co-transfected with two vectors into goat skeletal muscle satellite cell, and the normalized luciferase activity was assayed. The results are presented as the means ± SEM. Statistical significance was analysed by the T-test (*P* < 0.05).

Using target prediction software (TargetScan), we found a binding site for miR-27b in the *Pax3* 3′UTR (Fig. 10). In addition, the expression patterns of miR-27b and *pax3* during skeletal muscle satellite cell proliferation and differentiation were determined by qPCR. The results showed a negative correlation between the expression patterns of *pax3* and miR-27b. We also found that there may be a close and direct relationship between miR-27b and *pax3*. To verify this hypothesis, we constructed two dual-luciferase reporter vectors, which were showed in Fig. 10, that included wild type or mutant *Pax3*, respectively. Compared with the NC, the luciferase activity of the cells that were co-transfected with miR-27b mimics and wild-type dual-luciferase reporter vector was significantly decreased, but the co-transfection of miR-27b mimics with a mutant-type dual-luciferase reporter vector was not significantly different than miR-27b NC (Fig. 10). Thus, we concluded that *Pax3* was a direct target gene of miR-27b in goat skeletal muscle satellite cells.

**Figure 11:**
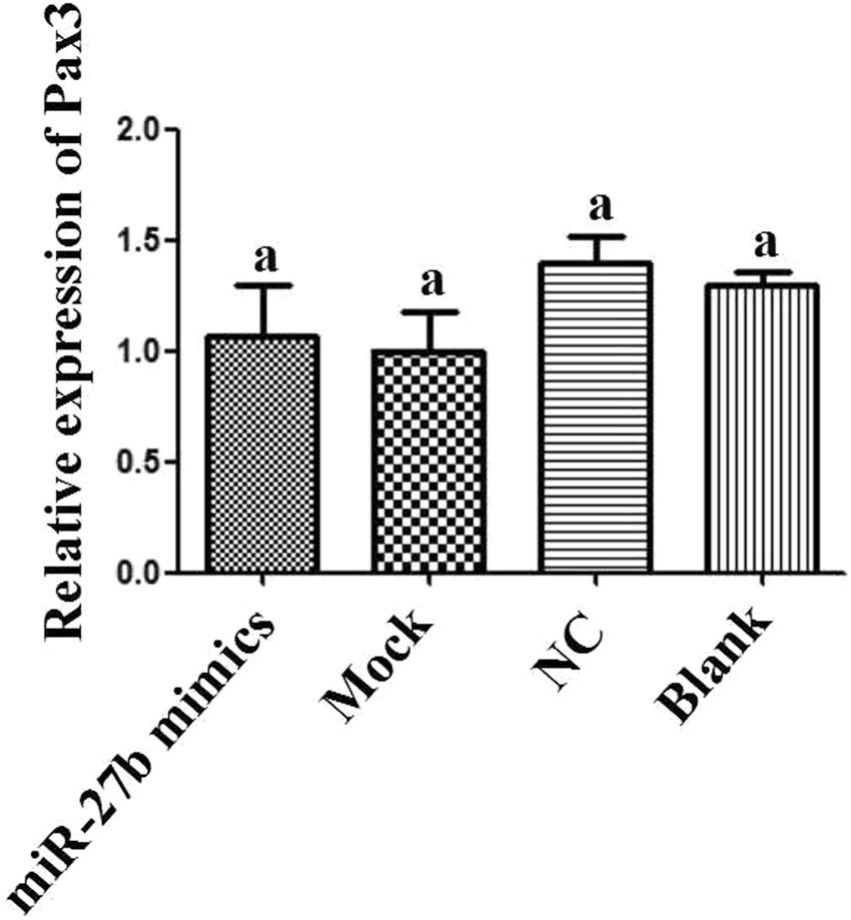
Expression of *Pax3* mRNA in cells transfected with miR-27b mimics. The data are presented as the mean ± standard error (n = 3), and bars with the same letter did not have significant difference (p < 0.05).

To further analyse the regulatory method, we used qPCR, immunofluorescence, and Western blot analysis to detect the expression of *Pax3* when the expression of miR-27b was verified. Using qPCR, we found that the expression of *Pax3* mRNA did not significantly differ between the over-expression miR-27b group and the NC (Fig. 11).

**Figure 12:**
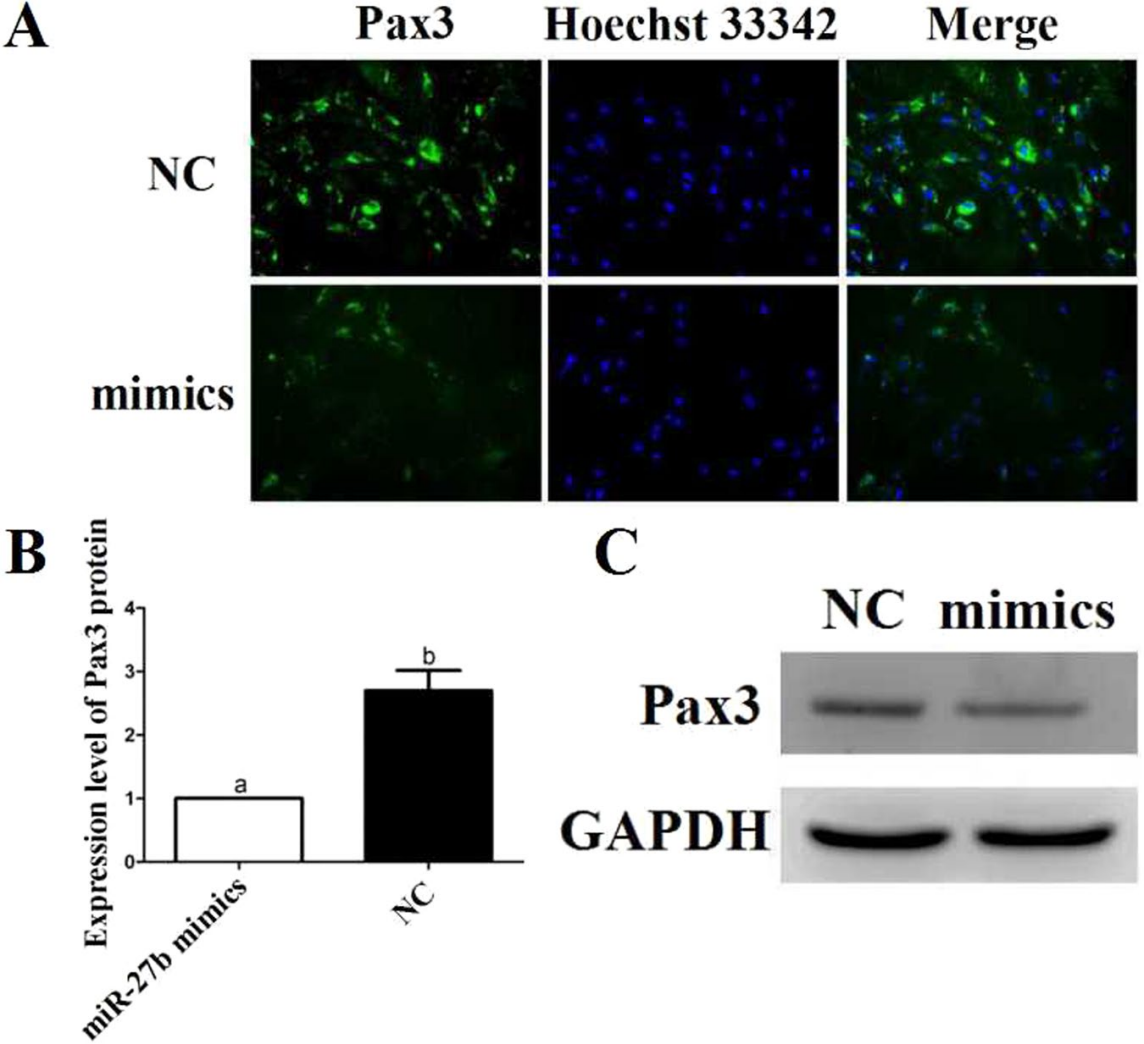
*Pax3* protein expression level after over-expression of miR-27b. (**A**) Immunofluorescence staining of Pax3 protein after over-expression of miR-27b. (**B**) The fluorescence intensity of Pax3 protein (µm^2^). (**C**) Western blot of Pax3 protein.

Immunofluorescence staining and western blot analysis were used to detect changes in the Pax3 protein level. When the expression of miR-27b was up-regulated in the cells, the fluorescence value and the signal for the blotting band (Figs S1 and S2) decreased (Fig. 12). Taken together, the results suggested that miR-27b could regulate the post-transcriptional expression of *Pax3* in goat.

## Discussion

Mutton is an important consumer product in daily life. However, the local goat breeds in China generally have problems, such as a small size, slow growth and low meat production, which severely constrain development of the mutton industry in China. Studies of the regulatory mechanisms of muscle growth and development at the molecular level can provide a theoretical basis for directional breeding for improved meat performance. miRNAs are endogenous noncoding RNAs that play a vital role in various biological processes, including the proliferation and differentiation of cells. As a member of the miR-27 family, miR-27b plays an important role in muscle development. It can inhibit skeletal muscle satellite cell proliferation, but it promotes differentiation^16^. Moreover, miR-27b has been reported to regulate *Pax3* protein levels, and this downregulation ensures rapid and robust entry into the myogenic differentiation programme in mice^16^. In our previous study, we found that miR-27b was expressed in a tissue-specific manner in the muscle of Anhuai goat^17^; however, its function and mechanism of action were unclear. In the present study, we first analysed the expression of miR-27b and its predicted target gene, *Pax3*, in the muscle tissue of Anhuai goat, and the same tissues of Boer goat were used as a reference control. We found that the expression of miR-27b in all collected muscle tissues of Anhuai goat, excluding kidney, was higher than in Boer goat, whereas the opposite expression pattern was observed for *Pax3*, which showed lower expression in Anhuai goat. Boer goat is famous for its high yield and rapid growth. Therefore, the results implied that the expression of *Pax3* was related to meat production in goat, however, the expression of miR-27b was negatively related to production.

To further explore the function of miR-27b and *Pax3* in muscle development in goat, the expression of the two transcripts in skeletal muscle satellite cells from different stages of proliferation and differentiation were analysed. The results showed that the expression of miR-27b gradually decreased with the proliferation of satellite cells, but increased with the differentiation of cells. However, the opposite trend was observed for *Pax3*, which indicated that miR-27b could promote the differentiation but inhibit the proliferation of muscle cells in goat. However, *Pax3* has a contradictory function. The transfection results further confirmed our hypothesis. Based on the EdU assay and detection of the expression of *MyoG* after up-regulating or down-regulating the expression of miR-27b, we demonstrated that miR-27b could inhibit the proliferation of goat skeletal muscle satellite cells. Moreover, a similar study of *Pax3* demonstrated the opposite phenomenon.

In previous studies, it has been shown that specific miRNAs play fundamental roles during muscle proliferation and differentiation by regulating a number of genes and transcription factors^18–20^. miR-101a is also up-regulated during the differentiation of goat skeletal muscle satellite cells, and it can promote satellite cell differentiation^21^. Some experiments have shown that miR-27a promoted myoblast proliferation by targeting myostatin and that miR-27a is up-regulated and its target gene myostatin down-regulated during C2C12 myoblast proliferation^22^. One study has shown that miR-27b participates in the regulation of the double-muscled phenotype in piedmontese cattle, and the results of a luciferase reporter assay demonstrated that bovine MSTN is a specific target of miR-27b and that miR-27b can promote skeletal muscle growth by targeting MSTN^23^. *Pax3*, as an upstream regulator of myogenesis, has been shown to play a vital role in muscle development^24^. The *Pax3* gene plays an important role in the growth and development of muscles, and *Pax3* expression maintains myogenic progenitors in an undifferentiated state and that the degradation of *Pax3* is essential for myogenic differentiation^25^.

Finally, we conducted a dual-luciferase reporter assay to assess whether *Pax3* is a target gene of miR-27b during the development of goat muscle. The results showed a lower fluorescence of wild-type than mutant-type. The qPCR, immunofluorescence staining, and Western blot analyses were used to determine the influence of miR-27b on the expression of *Pax3* mRNA and protein, respectively. We found that over-expression of miR-27b inhibited the protein, but not the mRNA, level of *Pax3*, which indicated that miR-27b could not inhibit *Pax3* transcription but could prevent Pax3 protein translation. Taken together, the results showed that miR-27b could regulate myogenic proliferation and differentiation by targeting *Pax3* in goat.

miRNAs are ~23-nucleotide-long, non-coding RNAs that are loaded into Argonaute (Ago) proteins. They provide binding specificity to Ago proteins, which they guide to specific elements complementary to the miRNA sequence in the 3′ untranslated region (UTR) of the mRNA. Upon binding to these elements, miRNAs repress target genes via a combination of translation repression and mRNA degradation^26^. However, in this study, miR-27b only inhibited *Pax3* gene translation and did not degrade *Pax3* mRNA. This phenomenon indicated that other regulatory mechanisms participate in the *Pax3* mRNA expression process.

In vertebrates, myogenic precursors originate from somites, proliferate and differentiate into myoblasts by undergoing several rounds of division and terminally differentiate into multi-nucleated myotubes^27,28^. The regulation of miRNAs during animal developmental processes is a key focus in the field of animal biology. It provides a new research strategy for people to study the regulation of animal development. miRNAs are extensively present in organisms and play an important role in life processes, including embryonic development, cell proliferation, cell apoptosis and lipid metabolism^29–32^. miRNAs can degrade mRNA or repress their translation when their own seed sequence binds to the target gene mRNA 3′ UTR region^33^. Many studies have confirming that miRNAs can play an important role in muscle growth and developmental processes. These processes are controlled through the coordination of myogenin, *MyoD*, *Myf5* and *Myf6*, which are well-known myogenic regulatory factors (MRFs)^34^. In addition, Sp1, a promoter of skeletal muscle differentiation, has been shown to induce *MyoD* activity and reduce cyclin-dependent kinase inhibitor 1 A (CDKN1A), which is a suppressor of cell proliferation^35^. miR-128 can regulate the proliferation and differentiation of bovine skeletal muscle satellite cells by repressing Sp1^36^. miR-1 and miR-133 originate from the same miRNA polycistronic region and are co-transcribed, but miR-1 and miR-133 have opposite functions, confirming that miRNAs may play a very important role in regulating the balance of cell proliferation and differentiation. Our research showed that miR-1 and miR-133 participated in skeletal muscle cell differentiation and maturation processes, in which miR-1 could promote the differentiation of skeletal muscle cells, but miR-133 inhibited differentiation to promote muscle cell proliferation^14^. One study found that miR-1 and miR-206, which are similar and have the same seed sequence, can facilitate bovine skeletal muscle satellite cell myogenic differentiation by restricting the expression of *Pax7* and *HDAC4*^37^.

In conclusion, our results demonstrated that miR-27b played an important role in goat skeletal muscle satellite cell proliferation and differentiation. We observed a lower expression of miR-27b in Boer goats than in Anhuai goats, in which it could promote the differentiation of skeletal muscle satellite cells but inhibit their proliferation by targeting *Pax3* gene. Research examining the mechanism of miR-27b in muscle growth can help to reveal the mechanisms underlying muscle development and provide a theoretical basis for animal breeding.

## Materials and Methods

### Experimental animals and cell culture

Based on a long-term observation, 6 adult female goats, including 3 Anhuai goats and 3 Boer goats, were selected for their muscle tissues. All experimental animals were raised on the farm of Boda Company (Hefei) under the unified management system of the field. The selected goats were dissected, and cardiac muscle, leg muscle, breast muscle, longissimus muscle, and kidney were collected rapidly and placed in 1.5-mL EP tubes without RNA enzyme and immediately cast into liquid nitrogen and then stored at −80 °C.

For the construction of cell lines, new-born Anhuai goats, which were also raised on the Boda company farm, were dissected. The dorsal longissimus muscle was digested in 0.1% collagenase for 40 minutes and then digested with 0.25% trypsin for 15 minutes. Isolated skeletal muscle satellite cells were subjected to a differential adhesion method for cell purification, in which after each 30-minute subculture, the cell suspension was re-seeded in a new culture dish (Corning, USA) for purification. Sixth-generation cells were selected and identified by immunofluorescent staining of Pax7 and MyoD1 proteins. The cells were cultured in growth medium containing 20% FBS (Gibco, USA) and DMEM/F12 (Hyclone, USA) at 37 °C in a 5% CO_2_ humidified atmosphere. When the cells grew to a density of 80–90%, the medium was removed and replaced with differentiation medium (2% FBS + 98% DMEM/F12). The morphological changes in the differentiated cells were observed under an inverted microscope, and the expression of the satellite cell differentiation marker *Myog* was detected by immunofluorescence and real-time quantitative PCR (qPCR). Satellite cells at the 7th, 10th, and 13th generation and fourth generation cells induced to differentiate for 1, 3, 5, and 7 days were collected for qPCR analysis. All experimental procedures involving goats performed in the present study had given prior approval by the ethics committee of Anhui Agricultural University under permit no. AHAU20101025, and all methods were performed in accordance with the relevant guidelines and regulations.

### RNA isolation and real-time quantitative PCR

The total RNA of muscle tissues and cells was extracted using the Total RNA kit II (OMEGA, USA), and 1% agarose gel electrophoresis and the A260/A280 and A260/A230 values were used to evaluate the integrity, concentration and purity of the RNA. For *Pax*3 quantification, first-strand complementary DNA was prepared from 1 µg total RNA from each sample using EasyScript One-step gDNA Removal and cDNA Synthesis SuperMix (TransGen, China). For miR-27b quantification, a stem-loop primer was designed for reverse transcription. qPCR was performed using an ABI 7500 Stepone Plus. The PCR mixture included 7.5 μL SYBR Real-time PCR System, 1.5 μL cDNA, 1 μL forward and reverse primers (10 μM). The cycle conditions were as follows: 95 °C for 10 mins, follows by 40 cycles of 95 °C for 15 s, 60 °C for 1 min, and 95 °C for 15 s. GAPDH and U6 were used as internal controls for mRNA and miRNA quantification, respectively. The expression levels of mRNA and miRNA were determined using the 2^-△△Ct^ method. Three biological replicates for each transcript were used, and the primers are listed in Table 1.

**Table 1:** The primers used for real-time quantitative PCR.

Function	Sequence (5′ → 3′)
Pax3 Forward	AGCCGCACCACCTTCACA
Pax3 Reverse	TCTGGGCCAGTTCCTCCC
MyoG Forward	CGTGGGCGTGTAAGGTGT
MyoG Reverse	GGCGCTCTATGTACTGGATGG
GAPDH Forward	CACAGTCAAGGCAGAGAAC
GAPDH Reverse	TACTCAGCACCAGCATCA
miR-27b stem loop primer	GTCGTATCCAGTGCGTGTCGTGGAGTCGGCAATTGCACTGGATACGACGCAGAAC
miR-27b Forward	GGGGTTCACAGTGGCTAA
miR-27b Reverse	TGAGGTGCTGTGCGTGAC
U6 Forward	CTCAGAATCACCCAATGC
U6 Reverse	ATGTTCATCCAGTTGTCAC

### Oligonucleotide transfection

The miR-27b mimics, inhibitor and negative control (NC) were all designed and synthesized by Ribobio (Ribobio, China). The small interfering RNA (siRNAs) targeting *Pax3* (si-Pax3) was designed and synthesized by GenePharma (GenePharma, China). The sense and antisense primers were 5′-GCCACAAGAUCGUGGAGAUTT-3′ and 5′-AUCUCCACGAUCUUGUGGCTT-3′. The *Pax3* CDS was synthesized by Generay (Generay, China) and inserted into the pLVX-IRES-Neo vector to form the pLVX-Pax3-FLAG-IRES-Neo plasmid. The miR-27b mimics, inhibitor, NC, si-Pax3, and pLVX-Pax3-FLAG-IRES-Neo were transfected into goat skeletal muscle satellite cells, respectively, using FugeneHD transfection reagent (Promega, USA) following the manufacturer’s manual. The expression levels of miR-27b and *Pax3* were detected using qPCR to determine the transfection efficiency.

### Cell proliferation and differentiation assay

Satellite cell proliferation was measured by the EdU (RiboBio, Guangzhou, China) cell proliferation assay according to the manufacturer’s protocol. First, goat skeletal muscle satellite cells were seeded in 96-well plates (4 × 10^3^−1 × 10^5^ cell/well) and cultured in GM to a normal growth stage after transfection in growth medium. Second, the medium was removed, and the cells were washed with PBS and incubated for 2 hours with medium containing 50 μM EdU. Immunostaining was then performed, and the fluorescence value was recorded using a TH4-200 inverted microscope (Olympus). The percentage of EdU-positive cells was calculated using ImageJ software. After transfection in growth medium, the cells were induced to differentiate in differentiation medium. In addition, the expression of *MyoG*, which is the marker gene of cell differentiation, was detected by qPCR to analyse the influence of miR-27b and *Pax3* on the differentiation of goat skeletal muscle satellite cells. The primers used for *MyoG* qPCR are listed in Table 1.

### Luciferase reporter assay

The primers Pax3-F and Pax3-R were used to PCR-amplify the 3′UTR of *Pax3* using Anhuai goat genomic DNA as template. TargetScan was used to predict the putative binding site of miR-27b on *Pax3*, and constructs with mutations in the putative binding site were generated by overlapping PCR using Pax3-F, Pax3-MR, Pax3-MF, and Pax3-R as primers and the product of the above PCR as template. The PCR and overlapping PCR products were subcloned into the pLUC dual-luciferase reporter vector (Promega, USA), respectively, to obtain the wild-type and mutational-type reporter plasmid (Pax3-WT and Pax3-MT). The primers used for plasmid construction are shown in Table 2.

**Table 2:** The primers used for the dual-luciferase report assay.

Name	Sequence
Pax3-F	CACAACTCGAGCCTGTTTCCGGTCTCCACAG
Pax3-R	AAGGATCCGGCTGCAACACGAAGATAGC
Pax3-MF	TGCAATTGACACTACCTCACGGATGCTTTGG
Pax3-MR	GAGGTAGTGTCAATTGCACTCTGATAAGCAGC

Notes: Underlined characters in *Pax*3-F and *Pax*3-R denote XhoI and BamHI restriction enzyme sites.

The satellite cells (2 × 10^4^) were seeded in 96-well plates and transfected with 0.2 µg of reporter vectors, 4.5 µg miR-27b mimics or negative control, and 30 µl Opti-MEM medium (Gibco, USA), using FugeneHD reagent (Promega, USA). Forty-eight hours later, the cells were collected for luciferase activity assays using the luciferase reporter assay system (Promega, USA).

### Immunofluorescence and Western blot analysis

For immunofluorescence, goat skeletal muscle satellite cells, which were identified or transfected with miR-27b mimics or NC, were fixed in 4% paraformaldehyde for 10 min, treated with 0.5% Triton X-100 at 37 °C for 15 min, blocked with 1% BSA for 1 h at room temperature, and incubated with primary antibodies against Pax7, MyoD1, Myog and Pax3 overnight and then with secondary antibodies. The nuclei were stained with Hoechst 33342 (RiboBio) for 30 min. The change in fluorescence value was evaluated using an inverted fluorescence microscope. Satellite cells that were only transfected with transfection agents were used as a blank control.

For western blot, the goat skeletal muscle satellite cells, which were transfected with miR-27b mimics or NC, were cultured for 48 h, and the total protein of the cells were extracted by the RIPA buffer (TaKaRa, Dalian, China) with 1% PMSF (protease inhibitor). The total protein added with the 6x SDS sample buffer was boiled at 100 °C for 5 min, and was separated by SDS-PAGE, transferred into the membrane. The transferred membrane was incubated overnight at 4 °C with primary antibodies for Pax3 (Cell Signaling, USA, 1:1000–1:3000 dilution) and GAPDH (Cell Signaling, USA, 1:1000–1:3000 dilution), and incubated with HRP-labled antiRabbit IgG secondary antibody (Abbkine, USA, 1:50–1:1000) for 1 h at 37 °C. At last, the result was analyzed by images software.

### Bioinformatics and statistical analyses

All data expressed as the mean ± SEM were compared by one-way ANOVA using SPSS software. Differences were regarded as significant at P < 0.05.

## Electronic supplementary material

Supplementary information
