# Supplementary material for: miR-27b regulates myogenic proliferation and differentiation by targeting Pax3 in goat
Source: Sci Rep. 2018 Mar 2;8:3909. doi: 10.1038/s41598-018-22262-4 (PMC5834623; doi:10.1038/s41598-018-22262-4)
Supplement: Supplementary file 1 — Supplementary information [file 41598_2018_22262_MOESM1_ESM.pdf]

miR-27b regulates myogenic proliferation and differentiation by targeting *Pax3* in goat

Ying-Hui Ling[1,2†], Meng-Hua Sui[1,2†], Qi Zheng[1,2†], Kang-Yan Wang[1,2], Hao Wu[1,2], Wen-Yong Li[4], Yong Liu[4], Ming-Xing Chu[5], Fu-Gui Fang[1,2]& Li-Na Xu[1,3*]

[1]College of Animal Science and Technology, Anhui Agricultural University, Anhui Hefei, China.

[2]Local animal genetic resources conservation and biobreeding laboratory of Anhui province, Anhui Hefei, China.

[3]Institute of Plant Protection and Agro-Products Safety, Anhui Academy of Agricultural Sciences, Hefei, Anhui 230031, China.

[4]Key Laboratory of Embryo Development and Reproductive Regulation of Anhui Province, Fuyang Normal University, Fuyang, Anhui, 236037,China.

[5]Key Laboratory of Farm Animal Genetic Resources and Germplasm Innovation of Ministry of Agriculture, CAAS, Beijing 100193, China.

[†] These authors contributed equally to this work.

*Corresponding author: Lina Xu: xulina26@hotmail.com.

Supplementary information

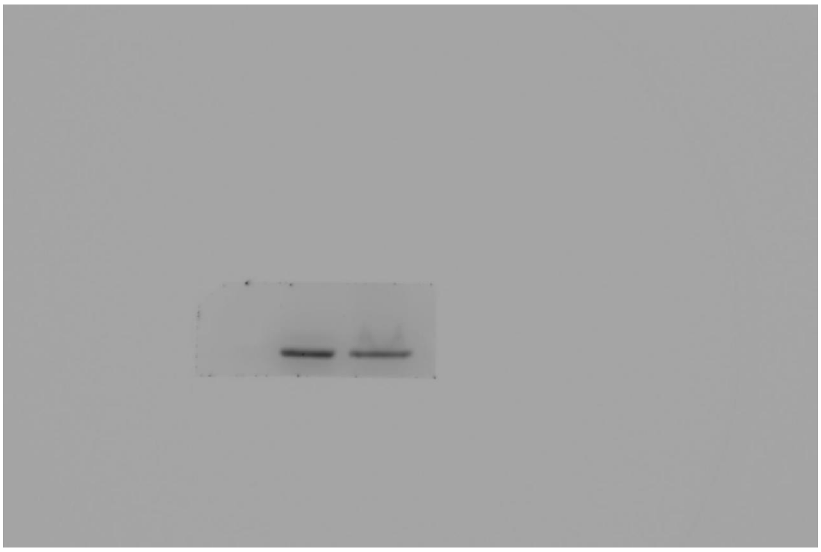

Figure S1. Western blot of Pax3 protein

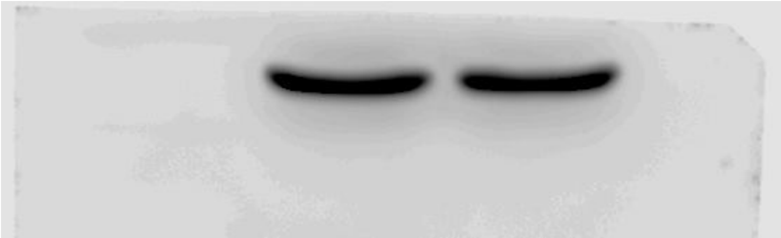

Figure S2. Western blot of GAPDH
